# Analysis of Alternative Splicing During the Combinatorial Response to Simultaneous Copper and Iron Deficiency in Arabidopsis Reveals Differential Events in Genes Involved in Amino Acid Metabolism

**DOI:** 10.3389/fpls.2022.827828

**Published:** 2022-01-31

**Authors:** Estefania Mancini, Antoni Garcia-Molina

**Affiliations:** ^1^Centre for Genomic Regulation, Barcelona, Spain; ^2^Centre for Research in Agricultural Genomics (CRAG), CSIC-IRTA-UAB-UB, Barcelona, Spain

**Keywords:** alternative splicing, copper deficiency, iron deficiency, combinatorial stress, systems biology, *Arabidopsis thaliana*

## Abstract

Copper (Cu) and iron (Fe) constitute fundamental nutrients for plant biology but are often limited due to low bioavailability. Unlike responses to single Cu or Fe deprivation, the consequences of simultaneous Cu and Fe deficiency have not yet been fully deciphered. Previously, it was demonstrated that Cu and Fe deficiency applied in combination imposes transcriptome, proteome, and metabolome changes different from those triggered under each deficiency individually. Here, we evaluated the effect of alternative splicing (AS) on the transcriptome of rosette leaves under single and simultaneous Cu and Fe deficiency. Differentially spliced genes (DSGs) and differentially expressed genes (DEGs) coincided in number (2,600 approx.) although the overlapping fraction was minimal (15%). Functional annotation of changes exclusively detected under simultaneous Cu and Fe deficiency revealed that DEGs participated in general stress responses and translation, while DSGs were involved in metabolic reactions, especially amino acid biosynthesis. Interestingly, transcripts encoding central features for tryptophan (Trp) and asparagine (Asn) synthesis – two significantly altered metabolites under simultaneous Cu and Fe deficiency – underwent exclusive intron retention events under the double deficiency. However, transcript and protein amounts for these enzymes did not correlate with Trp and Asn concentration. In consequence, we propose that AS might act as a regulatory mechanism to modify the stability and/or functionality of the enzymes and therefore fine-tune amino acid production during the combinatorial response to simultaneous Cu and Fe deficiency.

## Introduction

Copper (Cu) and iron (Fe) are essential nutrients for plants because they possess suitable redox properties to sustain fundamental processes such as photosynthesis, respiration, and antioxidative defense reactions, among others (Puig et al., 2007; Ravet and Pilon, 2013; Zhang, 2015). However, the same redox properties lead to cytotoxicity when Cu and Fe are present in excess (Puig et al., 2007; Ravet and Pilon, 2013). In consequence, plants require fine-tuned mechanisms to keep Cu and Fe levels within balanced ranges. Despite being abundant in soils, Cu and Fe bioavailability is often restricted due to the chelating effect of organic matter, low insolubility in alkaline soils, or overexploitation of farmlands for agricultural purposes (Morrissey and Guerinot, 2009; Puig and Penarrubia, 2009).

Plant responses to individual Cu or Fe scarcity have been extensively investigated, especially in the model plant *Arabidopsis thaliana* (hereafter Arabidopsis). The transcription factors SQUAMOSA PROMOTER BINDING PROTEIN-LIKE 7 (SPL7) and FER-LIKE IRON DEFICIENCY INDUCED TRANSCRIPTION FACTOR (FIT1) orchestrate the transcriptional reprogramming under Cu and Fe limitation (Colangelo and Guerinot, 2004; Yamasaki et al., 2009; Bernal et al., 2012). SPL7 and IRT1 promote high-affinity uptake systems consisting of FERRIC REDUCTION OXIDASE (FRO) metalloreductases to reduce Cu^2+^ and Fe^3+^ to Cu^+^ and Fe^2+^ prior to transport via COPPER TRANSPORT PROTEINS (COPTs) and IRON-REGULATED TRANSPORTER 1 (IRT1), respectively. In addition, the dissection of the mechanism of action of SPL7 and FIT1 unveiled points of cross-talk between Cu and Fe homeostatic networks. For instance, SPL7 triggers the replacement of Cu/Zn-depending SUPEROXIDE DISMUTASE by the Fe-depending isoform to prioritize Cu ions in chloroplasts for PLASTOCYANINE and, thus, preserve photosynthesis under Cu deficient conditions (Yamasaki et al., 2009; Bernal et al., 2012). On the other hand, FIT1 promotes FRO4/5 and COPT2 to rely on Cu-depending proteins when Fe is limiting (Colangelo and Guerinot, 2004).

However, our understanding of the molecular mechanisms involved in combinatorial responses to simultaneous Cu and Fe deficiency is still limited. This fact is of special relevance as molecular responses to combinatorial stress trigger patterns that largely differ from those activated under each stressor individually (Atkinson et al., 2013; Prasch and Sonnewald, 2013; Rasmussen et al., 2013; Suzuki et al., 2014; Gupta et al., 2016). Recently, a systems biology study provided a holistic picture of transcriptome, proteome, and metabolome changes under single and simultaneous Cu and Fe deficiencies in Arabidopsis adult leaves (Garcia-Molina et al., 2020). Quantitatively, the combinatorial response caused half of all detected transcripts and proteins that significantly changed in abundance. Conditional networks on molecular changes under single and double Cu and Fe deficiencies displayed important divergences in topology and detection of communities, revealing substantial differences in co-expression of biological processes. Indeed, data mining on changing transcripts and proteins as a result of the interaction between Cu and Fe deficiencies uncovered specific expression patterns for transcripts related to ribosome subunit conformation and translation activities, and for proteins assisting protein folding and degradation. At the metabolome level, the combinatorial response to double Cu and Fe deficiency provoked decreases in the total fraction of sugars and organic acids from the tricarboxylic acid cycle in comparison with single deficiencies, and limited the increase in amino acids observed under single Fe deficiency. Further analysis of metabolome profiles explained such trends by significant drops in the concentration of phenylalanine (Phe), asparagine (Asn), tryptophan (Trp), and the organic acid fumarate. Moreover, loss-of-function lines of the cytosolic fumarase FUMARASE2 displayed improved growth and photosynthesis performance in seedlings cultivated under Cu and Fe deficient conditions, which was accompanied by balanced levels of amino acids (Garcia-Molina et al., 2021). This finding confirmed the existence of specific mechanisms exclusively operating in plants under simultaneous Cu and Fe deficiency and posed fumaric acid as a central modulator of the combinatorial response.

Alternative splicing (AS) is a post-transcriptional mechanism specific to eukaryotic organisms consisting in the differential processing of the exons and introns in precursor mRNAs to generate different isoforms of the same transcript. In plants, it is estimated that AS affects up to 70% of all precursor mRNAs, encompassing multiple exons with intron retention being the most predominant event (Filichkin et al., 2010; Marquez et al., 2012). The resulting transcript isoforms can display altered stability, translational efficiency, or subcellular retention, or can be translated into protein variants with divergent structures and functions. Thus, AS acts as a central regulatory strategy to sustain plant growth and development under different conditions (Reddy et al., 2013; Laloum et al., 2018; Szakonyi and Duque, 2018; Chaudhary et al., 2019). Moreover, recent studies in the field contributed in demonstrating the relevance of AS events in transcriptome responses to abiotic stress conditions as well as part of the so-called “memory stress” program (Ding et al., 2014; Calixto et al., 2018; Kannan et al., 2018; Ling et al., 2018; Huang et al., 2019).

How AS is involved in the combinatorial response to simultaneous Cu and Fe deficiency still remains an open question. Therefore, in this work we aimed to extend the holistic picture of molecular changes in response to the double Cu and Fe deficiency with the contribution of AS in transcript regulation. Our analysis of the transcriptome data generated in Garcia-Molina et al. (2020) uncovered a specific cohort of genes exclusively undergoing AS during the combinatorial response. These genes were related to biosynthetic pathways for amino acids changing under this condition, especially Asn and Trp. Further integration of transcriptome, proteome, and metabolome profiles revealed lack of correlation between transcripts and proteins for enzymes synthesizing Trp and Asn and metabolite concentration when both Cu and Fe are limiting. Accordingly, we propose that AS events might act as a potential regulatory mechanism to adjust metabolic composition to plant demands during the combinatorial response to double Cu and Fe deficit.

## Materials and Methods

### Data Collection

Transcriptome (GEO ID: GSE125894), proteome (Pride ID: PXD013598), and metabolome (Supplementary Table 1) profiles of adult rosette leaves reported by Garcia-Molina et al. (2020) were used in this study. Briefly, 12-day-old *A. thaliana* (ecotype Col0) seedlings grown on soil were transferred to hydroponic cultures with full 1/10 Hoagland medium (Hermans et al., 2004) for 12 days and then treated with the same medium or without Cu and/or Fe salts for 10 days. RNA-seq libraries (*n* = 2 independent replicates per treatment) were sequenced using standard Illumina HiSeq 2500 (Illumina, San Diego, CA, United States) protocols. Proteomes (*n* = 4 independent replicates per treatment) were profiled by means of liquid chromatography-tandem mass spectrometry (LC-MS/MS) and processed with MAXQUANT (Cox and Mann, 2008). Metabolite concentration (*n* = 4 and 7 independent replicates per treatment at 5 and 10 days, respectively) was determined by targeted gas chromatograph coupled to a time-of-flight mass spectrometer (GC-TOF-MS) according to the Golm Metabolome Database (Kopka et al., 2005) using ribitol and ^13^C-sorbitol as internal standards for relative quantification.

### Analysis of Alternative Splicing

The analysis of alternative splicing was conducted in *R* using the *ASpli* package (Mancini et al., 2021). Sequencing reads derived from RNA-Seq files were mapped to the *A. thaliana* genome (TAIR10) using the aligner tool STAR (Dobin et al., 2012) with default parameters, except for maximum intron length set at 5,000 nucleotides. Then, the transcriptome was partitioned into subgenic regions named “bins” as proposed on DEXseq (Anders et al., 2012). Read counts in each bin region were recorded. Null values (NA) representing lack of coverage of bins were manually replaced by 0. Splice junction information was employed to compute the Percent of Inclusion (PSI) and Percent of Intron Retention (PIR) for each bin.

Significant changes in alternative splicing events were calculated from differences in PSI or PIR using a two-way ANOVA (*p*-value ≤ 0.05) for the interaction between Cu and Fe deficiencies. Subsequently, significant changes in alternative splicing events among conditions were declared using an absolute log_2_ fold-change for PSI or PIR ≥ 1 in pair-wise comparisons (adjusted-*p*-value ≤ 0.05, Tukey’s *post hoc* test).

### Bioinformatic Tools

Data analysis and statistical treatments were conducted in *R*. Data transformation, Pearson correlations, and ANOVA were computed using the *stats* and *agricolae* package (available at The Comprehensive *R* Archive Network, CRAN).^1^ Heatmaps with hierarchical clustering were elaborated using the *pheatmap* package (available at CRAN; see text footnote 1). Significant enrichment in Gene Ontology terms (adjusted-*p*-value ≤ 0.05, Fisher’s exact test) was carried out in *Thalemine*^2^ and redundant terms were removed in *REVIGO*^3^ to obtain a small list (0.5) (Supek et al., 2011). Benjamini-Hochberg’s method was applied for multiple testing correction in all cases. To identify metabolic reactions targeted by alternative splicing events, differentially spliced genes were loaded onto *Mapman* v.3.5.1^4^ and the bins for amino acid reactions in the category “metabolism overview” extracted (Thimm et al., 2004). Alternative splicing events were visualized in *Integrative Genome Viewer* (Robinson et al., 2011) according to the coverage in the RNA-Seq experiments using log_10_-transformed read counts.

## Results

### Alternative Splicing Events During the Combinatorial Copper and Iron Deficiency Target a Fraction of Genes That Are Not Differentially Expressed

**FIGURE 1:**
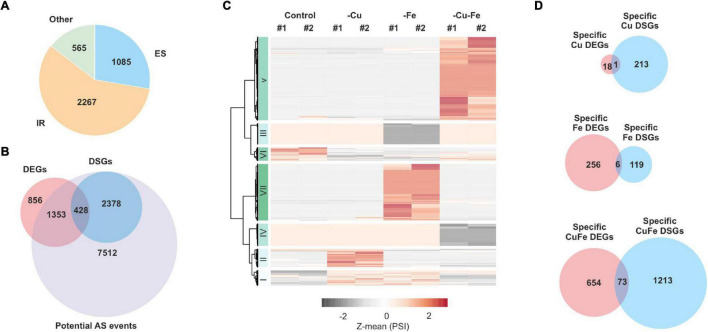
Analysis of differential splicing events under individual and simultaneous copper and iron deficiency. RNA-Seq read counts covering exonic and intronic regions from the transcriptome profiles of rosette leaves treated for single and double copper and iron deficiency reported in Garcia-Molina et al. (2020) were obtained with the *ASpli* package (see “Materials and Methods”). **(A)** Classification of AS events into intron retention (IR), exon skipping (ES) or other events (Other). **(B)** Euler diagram comparing the fraction of all genes potentially affected by AS, the differentially spliced genes (DSGs) detected and differentially expressed genes (DEGs) reported in Garcia-Molina et al. (2020). **(C)** Heatmap with hierarchical clustering according to the Ward D2 method with Z-mean for significant changes in percentage of intron retention or bin inclusion (represented as PSI) (*p*-value ≤ 0.05, two-way ANOVA) under standard conditions (control), copper (–Cu) and iron (–Fe) deficiency or double deficiency (–Cu–Fe). **(D)** Euler diagrams comparing the fraction of specific DEGs in Garcia-Molina et al. (2020) and specific DSGs for each treatment. Specific DEGs and DSGs were defined as the common fraction in multiple comparisons among significant changes in each of the conditions (absolute fold-change ≥ 2 in pair-wise comparisons, adjusted-*p*-value ≤ 0.05, Tukey’s test; Supplementary Figure 1).

High-throughput profiling and systemic analysis of the transcriptome, proteome, and metabolome of adult rosette leaves grown under double Cu and Fe deficiency for 10 days uncovered an array of molecular changes that were not triggered under single Cu or Fe deficiencies (Garcia-Molina et al., 2020). To complete this picture with the contribution of AS in the combinatorial response to simultaneous Cu and Fe deficiency, the transcriptome profiles generated in Garcia-Molina et al. (2020) were analyzed using the *ASpli* package (Mancini et al., 2021). Reads spanning intronic and exonic bins as well as junctions were used to estimate the PIR or PSI (Mancini et al., 2021) under each of the conditions. Differential alternative splicing events (DASs) were declared based on significant changes in PIR or PSI for the interaction between Cu and Fe deficiencies (*p*-value ≤ 0.05, two-way ANOVA). In total, 3,917 DASs were identified −27.7% of these corresponded to annotated exon skipping (ES) events, whereas 57.8% were intron retention (IR) events and 14.40% were other minoritarian forms of AS events. Moreover, DASs were located in 2,806 different genes that were further considered differentially spliced genes (DSGs) (Figures 1A,B).

Interestingly, although the DSG fraction represented 33.56% of all potential DSGs annotated in *ASpli*, however, only 428 DSGs (15.25% of all DSGs) were previously reported as differentially expressed genes (DEGs) in Garcia-Molina et al. (2020; Supplementary Table 2) using the same cut-off for significance. Similarly, only 16.23% of the reported DEGs underwent significant AS events (Figure 1B). This observation indicates that transcriptome rewiring by AS during single and simultaneous Cu and Fe deficiency targets a coreset of transcripts unchanged as regards of abundance.

To identify expression patterns in the effect of AS on the transcriptome, DAS were used to elaborate a heatmap with hierarchical clustering. As depicted in Figure 1C, seven main clusters of DASs were detected using the Ward D2 method. With the exception of the two minor clusters I and VI (486 AS events, 12.04%) that represented DAS under both single deficiencies, the rest of the clusters included AS events specific to each of the conditions, i.e., cluster II (311 AS events, 7.93%) contained changes mainly taking place during Cu deficiency, clusters III and VII (1,323 AS events, 33.77%) changes only occurring under Fe deficiency, and clusters IV and V (1,801 AS events, 45.97%) changes specific to double Cu and Fe deficiency. Another remarkable trend was that the main fraction of all identified DAS (68.94%) was motivated by increases in AS rates (Figure 1C).

Since our analysis unveiled that DAS was mainly treatment-specific, the fraction of genes that specifically underwent AS events under each of the treatments was captured by means of multiple comparisons of DAS that displayed an absolute fold-change of at least two in pair-wise comparisons (adjusted-*p*-value ≤ 0.05, Tukey’s test; Supplementary Figure 1 and Supplementary Table 3). Thus, 214 specific Cu-DSGs, 125 specific Fe-DSGs, and 1,286 specific CuFe–DSGs were identified. Although Cu deficiency alone had a low impact on transcriptome changes, the number of Cu–DSGs was twice that of Fe–DSGs (Supplementary Figure 1). The existence of a combinatorial effect in simultaneous Cu and Fe deficiency was also demonstrated at the AS level since the number of specific CuFe–DSGs was four-fold higher than the sum of those found under both single deficiencies. Again, a small overlap between specific DSGs and specific DEGs per condition – quantified as less than 10% of DEGs being DSGs – was found (Figure 1D), further confirming that changes in transcript abundance were not motivated by significant splicing events.

### Specific Alternative Splicing Events Under the Combinatorial Response to Copper and Iron Deficiency Target Biosynthetic Pathways for Changing Amino Acids

**FIGURE 2:**
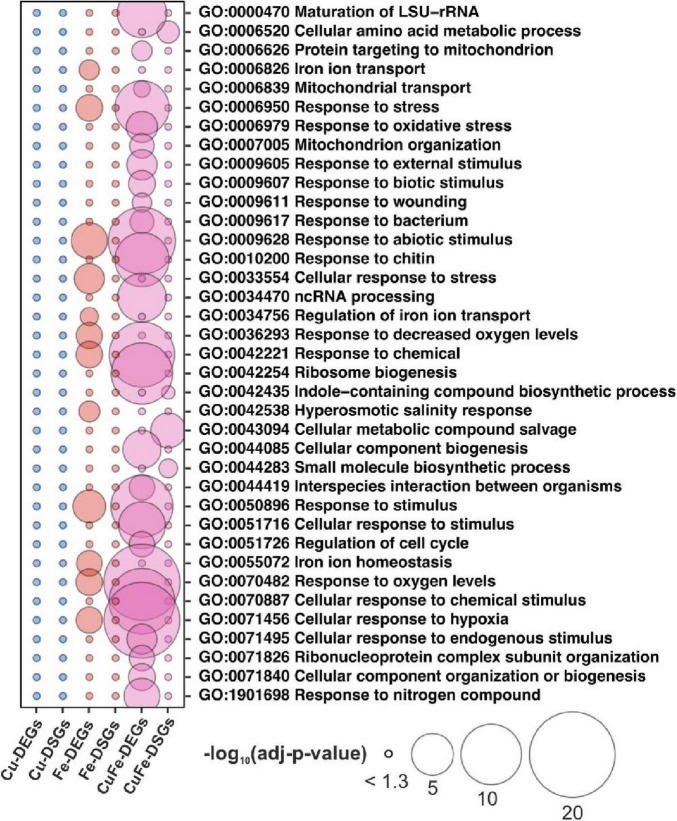
Functional annotation of specific changes in transcriptome and alternative splicing under single and simultaneous copper and iron deficiency. Bubble plot depicting differences in specific DEGs and DSGs detected under single and double copper and iron deficiency treatments (Cu, Fe, and CuFe, respectively). Specific DEGs and DSGs were analyzed for significant gene ontology (GO) term enrichment (adjusted-*p*-value ≤ 0.05, Fisher’s test) and filtered for redundancy by REVIGO (filter 0.5). The size of the bubble is proportional to the –log10-transformation of the adjusted-*p*-value in the Fisher’s test.

Given the divergence in the coreset of genes that experienced changes in AS and transcript abundance, we interrogated whether different biological processes would be regulated in each case. To this end, functional annotation of specific DEGs and DSGs was conducted by significant enrichment of non-redundant gene ontology (GO) terms (adjusted-*p*-value ≤ 0.05, Fisher’s exact test, and filter 0.5 in REVIGO, Supek et al., 2011). Cu deficiency did not lead to significant enrichments of neither DSGs nor DEGs, due to the low impact of the treatment, whereas Fe-DEGs rendered enrichment in 12 terms mainly related to Fe homeostasis (“Iron ion homeostasis,” “Iron ion transport,” or “Regulation of iron ion import”), hypoxia (“Response to oxygen levels,” “Response to decreased oxygen levels”), and abiotic stress response (“Response to abiotic stimulus”) (Figure 2). However, 26 and four non-redundant terms were found for CuFe–DEGs and CuFe-DSGs, respectively (Figure 2). CuFe–DEGs were related to general responses to both biotic and abiotic stress, (“Response to stress,” “Response to abiotic stimulus,” “Response to biotic stimulus,” “Response to bacterium”), translation (“Ribosome biogenesis,” “Maturation of LSU-rRNA”, “Ribonucleoprotein complex subunit organization”), nitrogen homeostasis (“Response to nitrogen compound”), mitochondrion biology (“Mitochondrion organization,” “Mitochondrial transport”), or cell cycle (“Regulation of cell cycle”). On the other hand, CuFe-DSGs participate in pathways for compound salvage and amino acid metabolism, among others (Figure 2). These observations indicate that the reprogramming of the majority of biological processes involved in the response to single Fe and double Cu and Fe deficiencies takes place via changes in transcript abundance. Furthermore, our data suggest that DSGs detected under single Cu and Fe deficiencies might be the consequence of either the necessity to fine-tune isolated sets of transcripts, or aberrant splicing events due to stress imposed by the treatments. In contrast, AS events under simultaneous Cu and Fe deficiency might rather obey a more general regulatory strategy aimed at adjusting plant amino acid composition.

Previously, it was shown that coincident Cu and Fe limitation imposed a particular reconfiguration of the amino acid fraction in adult leaves, and how seedlings tolerant to the double deficiency via suppression of the cytosolic *FUMARASE2* restored amino acid levels to those under standard conditions (Garcia-Molina et al., 2020, 2021). Due to the potential prevalence of targeting pathways related to amino acid metabolism and the relevance of amino acids in plant homeostasis during simultaneous Cu and Fe deficiency, CuFe–DSGs were mapped into *Mapman* bins for the category “central metabolism” (Thimm et al., 2004) to gain more insight as to which amino acid reactions were affected by AS events. In this way, 28 of the 175 bins (Supplementary Table 4) for central metabolism mapped by specific CuFe–DSGs were involved in biosynthetic reactions for the aromatic amino acid Trp, for the group of serine-glycine-cysteine, or for the Asp family, as well as catabolic reactions for the branched-chained amino acids leucine and isoleucine (Table 1). On the other hand, CuFe–DEGs mapped into five bins related to amino acid metabolism and only overlapped with CuFe–DSGs for *ASPARTATE AMINOTRANSFERASE 2* (*ASP2*) (Table 1).

**TABLE 1:** Identified Mapman bins related to amino acid metabolism.

Description of the bin	DEG	Description of the gene
**Amino acid metabolism.synthesis**		
Aspartate.aspartate aminotransferase	AT5G19550	ASPARTATE AMINOTRANSFERASE 2 (ASN2)
Aspartate.aspartate aminotransferase	AT1G72330	ALANINE AMINOTRANSFERASE 2 (ALAAT2)
Serine-glycine-cysteine group.serine.phosphoserine aminotransferase	AT3G19030	Unknown
Serine-glycine-cysteine group.cysteine	AT5G65720	Alanine-glyoxylate aminotransferase, putative
Serine-glycine-cysteine group.cysteine.OASTL	AT4G14880	*O*-ACETYLSERINE (THIOL) LYASE ISOFORM A1 (OASA1)
**Amino acid metabolism.degradation**		
Glutamate family.glutamine	AT5G13780	*N*-TERMINAL ACETYLTRANSFERASE 10 (NAA10)

**Description of the bin**	**DSG**	**Description of the gene**

**Amino acid metabolism.synthesis**		
GABA.Glutamate decarboxylase	AT1G65960	GLUTAMATE DECARBOXYLASE 2 (GAD2)
Aspartate.aspartate aminotransferase	AT5G19550	ASPARTATE AMINOTRANSFERASE 2 (ASP2)
Alanine.alanine aminotransferase	AT1G70580	ALANINE-2-OXOGLUTARATE AMINOTRANSFER. 2 (AOAT2)
Alanine.alanine-glyoxylate aminotransferase	AT2G13360	ALANINE:GLYOXYLATE AMINOTRANSFERASE (AGT)
Glutamate family.proline.d 1-pyrroline-5-carboxylate synt	AT2G39800	DELTA1-PYRROLINE-5-CARBOXYLATE SYNTH. 1 (P5CS1)
Aspartate family.asparagine.asparagine synthetase	AT5G65010	ASPARAGINE SYNTHETASE 2 (ASN2)
Aspartate family.methionine	AT4G34840	METHYLADENOSINE NUCLEOSIDASE 2 (MTN2)
Aspartate family.methionine.homocysteine S-methyl	AT3G22740	HOMOCYSTEINE S-METHYLTRANSFERASE 3 (HMT3)
Aspartate family.methionine.homocysteine S-methyl	AT1G78140	Methyltransferase-related
Aspartate family.lysine.diaminopimelate epimerase	AT3G53580	Diaminopimelate epimerase family protein
Aspartate family.misc.homoserine.bifunctional aspartate kinase/homoserine dehydrogenase	AT4G19710	Aspartate kinase/homoserine dehydrogenase, putative
Aspartate family.misc.homoserine.aspartate semialdehyde dehydrogenase	AT1G14810	Semialdehyde dehydrogenase family protein
Branched chain group.leucine specific.3-isopropylmalate dehydrogenase	AT5G14200	3-Isopropylmalate dehydrogenase, putative
Serine-glycine-cysteine group.glycine.glycine transamin	AT1G70580	ALANINE-2-OXOGLUTARATE AMINOTRANSFER. 2 (AOT2)
Serine-glycine-cysteine group.glycine.serine glyoxylate aminotransferase	AT2G13360	ALANINE:GLYOXYLATE AMINOTRANSFERASE (AGT)
Serine-glycine-cysteine group.cysteine.OASTL	AT3G22460	O-ACETYLSERINE (THIOL) LYASE ISOFORM A2 (OASA2)
Serine-glycine-cysteine group.cysteine.SAT	AT2G17640	SERINE O-ACETYLTRANSFERASE (ATSERAT3;1)
Aromatic aa.tryptophan.phosphoribosyanthranilate isom	AT5G05590	PHOSPHORIBOSYLANTHRANILATE ISOMERASE 2 (PAI2)
Aromatic aa.tryptophan.phosphoribosyanthranilate isom	AT1G07780	PHOSPHORIBOSYLANTHRANILATE ISOMERASE 1 (PAI1)
Aromatic aa.tryptophan.indole-3-glycerol phosphate synt	AT5G48220	Indole-3-glycerol phosphate synthase, putative (InGPS)
Aromatic aa.tryptophan.tryptophan synthase	AT5G54810	TRYPTOPHAN SYNTHASE BETA-SUBUNIT 1 (TSB1)
Aromatic aa.tryptophan.tryptophan synthase	AT4G02610	Tryptophan synthase, alpha subunit, putative (TSA1)
Histidine.glutamine amidotransferase/cyclase	AT4G26900	IMIDAZOLEGLYCEROL-PHOSPHATE SYNTAHSE (HISN4)
**Amino acid metabolism.degradation**		
Aspartate family.asparagine.L-asparaginase	AT5G08100	L-Asparaginase/L-asparagine amidohydrolase (ASPGA1)
Branched-chain group.shared	AT3G08860	Alanine-glyoxylate aminotransferase, putative (PYD4)
Branched-chain group.leucine	AT4G34030	3-METHYLCROTONYL-COA CARBOXYLASE (MCCB)
Branched chain group.isoleucine	AT5G48880	PEROXISOMAL 3-KETO-ACYL-COA THIOLASE 2 (KAT5)
Aromatic aa.tryptophan	AT5G65940	BETA-HYDROXYISOBUTYRYL-COA HYDROLASE 1 (CHY1)

*Specific differentially expressed genes (DEGs) and differentially spliced genes (DSGs) detected in the double deficiency were mapped onto the category “central metabolism” in Mapman. Bins were sorted for synthesis or degradation and presented with the bin description, the gene ID, and the description of the gene.*

**FIGURE 3:**
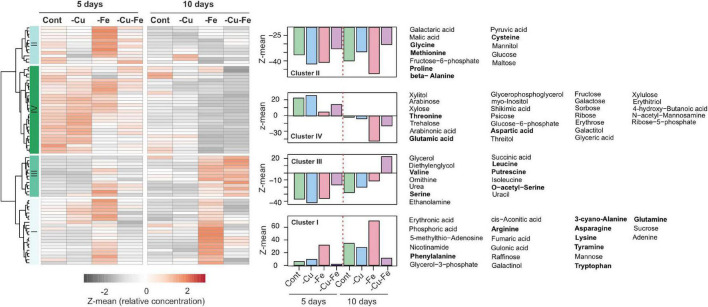
Metabolome profiles in response to single and double deficiency of copper and iron. Heatmap with hierarchical clustering according to the Ward method with Z-means for the median of the relative concentration of metabolites from rosette leaves exposed to standard conditions (control, Cont), copper (–Cu), and iron (–Fe) deficiency or double deficiency (–Cu–Fe) for 5 (*n* = 4) or 10 days (*n* = 7) in Garcia-Molina et al. (2020; Supplementary Table 1). For each cluster in the heatmap the trend is depicted as the average of the Z-mean of the compounds along with the name of the metabolites. Amino acids are highlighted in bold.

To address the relevance of AS changes in the amino acid composition of plants, we recovered the metabolome profiles in response to Cu, Fe, and the double Cu and Fe deficiency treatments after 5 and 10 days described in Garcia-Molina et al. (2020). Data were filtered for compounds with unknown mass and metabolites that were not present in all replicates. In this way, 76 metabolites were retained (Supplementary Table 1), and the median of normalized abundance was used to draw a heatmap with hierarchical clustering. Accordingly, four main patterns were identified (Figure 3). Metabolites in cluster I (22 metabolites) accumulated under control and Cu deficient conditions overtime and displayed a pronounced increase under Fe deficiency at both 5 and 10 days of treatment, while being decreased under simultaneous Cu and Fe deficiency (Figure 3). This pattern is of special relevance as it recapitulates the behavior of fumaric acid, adenine, the amino acids Asn, Phe, Trp and the Trp-derivative tyramine, which were previously identified as significantly altered metabolites (SAMs) in the combinatorial response to double Cu and Fe deficiency (Garcia-Molina et al., 2020). Compounds in cluster II (13 metabolites) rose in content after 5 days of Fe deficient treatment, but then remained steady under all conditions after 10 days. Cluster III (14 metabolites) included metabolites that increased under both Fe deficient and double Cu and Fe deficient treatments, with a more remarkable increase under double Cu and Fe deficiency (Figure 3). Finally, cluster IV (29 metabolites) contains, above all, sugars (trehalose, fructose, galactose, and sorbose) and sugar-alcohols (myo-inositol, xylitol, and galactitol), which decrease in content after 10 days under all treatments, with a more pronounced drop under both treatments lacking Fe (Figure 3).

Interestingly, cluster I – which contains the SAMs under simultaneous Cu and Fe deficiency as mentioned above – included the main fraction of amino acids that are end products of the metabolic reactions identified by *Mapman* bins using CuFe-DSGs, namely glutamine (Gln), Asn, and Trp, as well as the rest of the aromatic amino acids (Phe and tyrosine, Tyr), and tyramine (Figure 3). Consequently, we hypothesized that AS could operate as a new layer in the combinatorial response to refine transcriptome changes in order to adjust amino acid composition to plant necessities under the double Cu and Fe deficiency.

### Transcripts Encoding Enzymes Central to Tryptophan and Asparagine Biosynthesis Display Intron Retention Events Under Simultaneous Copper and Iron Deficiency

With the aim of dissecting the effect of AS on metabolic pathways for amino acids exclusively changing under the combinatorial Cu and Fe deficiency, we focused on those for Trp and Asn since they are two SAMs and the *Mapman* bins mapped by CuFe-DSGs allowed us to extract central features in their biosynthesis. This is the case of PHOSPHORIBOSYLANTHRANILATE ISOMERASE 1 and 2 (PAI1/2), INDOLE-3-GLYCEROL PHOSPHATE SYNTHASE (InGPS), TRYPTOPHAN SYNTHASE A (TSA1), and TRYPTOPHAN SYNTHASE BETA-SUBUNIT 1 (TBS1) for Trp (Table 1). For Asp, we found ASP2, the enzyme catalyzing the conversion of oxaloacetate to Asp in the first step of Asn biosynthesis, ASPARAGINE SYNTHETASE 2 (ASN2), the main enzyme mediating the condensation of Asp and Gln to render Asn, and L-ASPARAGINASE (ASPGA1), an enzyme that can degrade Asn to render Asp again (Table 1).

**FIGURE 4:**
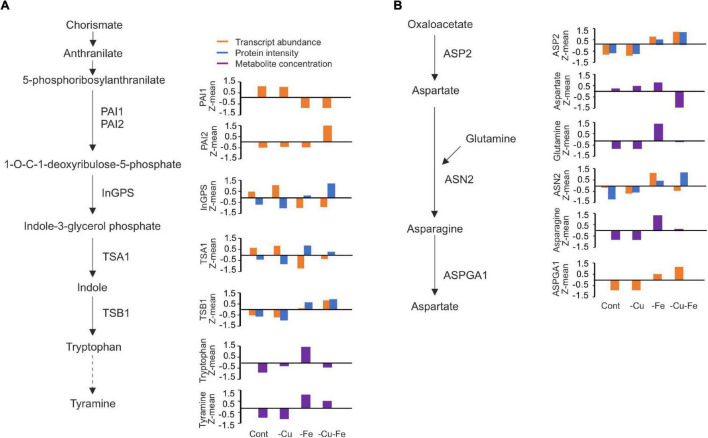
Molecular changes in tryptophan and asparagine biosynthetic pathways in response to single and double deficiency of copper and iron. **(A,B)** Overview of the metabolic reactions involved in tryptophan (Trp, **A**) and asparagine (Asn, **B**) biosynthesis. Depicted are the metabolic intermediates and the enzymes catalyzing each of the reactions. Z-means of transcript counts (Fragments Per Kilobase Million, FPKM), proteins (LC-MS chromatography peak intensity), and metabolite (normalized concentration to ^13^C-sorbitol and fresh weight) for each condition at 10 days, as reported in Garcia-Molina et al. (2020), were plotted. PAI1,2, PHOSPHORIBOSYLANTHRANILATE ISOMERASE 1 AND 2; InGPS, INDOLE-3-GLYCEROL PHOSPHATE SYNTHASE; TBS1, TRYPTOPHAN SYNTHASE BETA-SUBUNIT 1; TSA1, TRYPTOPHAN SYNTHASE A; ASP2, ASPARTATE AMINOTRANSFERASE 2; ASN2, ASPARAGINE SYNTHETASE 2; ASPGA1, L-ASPARAGINASE.

To first infer potential molecular mechanisms regulating Trp and Asn biosynthetic pathways, the transcriptome, proteome, and metabolome data generated in Garcia-Molina et al. (2020) were integrated as depicted in Figure 4. Regarding Trp biosynthesis, PAI1 and 2 showed antagonistic trends in transcript abundance under both Fe and double Cu and Fe deficient conditions, as *PAI1* levels dropped under both treatments, whereas those for *PAI2* increased (Figure 4A) – proteome profiles did not provide information at the protein level. Transcript and protein amounts of the remaining intermediate enzymes, InGPS, TSA1, and TBS1, displayed changes under both treatments lacking Fe, though not always coincident in trend (absolute *r* > 0.85; Table 2). Indeed, all changes at the protein level represented increases in abundance, whereas the InGPS and TSA1 transcript amounts decreased (*r* = −0.87 and −0.96) and those for TSB1 increased (*r* = 0.95; Figure 4A and Table 2). However, the pattern of transcript and protein amounts of the intermediate enzymes could not explain changes in Trp concentration (*r* < 0.65) due to the drop experienced under double Cu and Fe deficient conditions, except for TSA1 (*r* = −0.99 for the transcript and 0.76 for the protein; Figure 4A and Table 2). However, a better correlation between transcripts and proteins of intermediate enzymes and tyramine concentration – the degradative product of Trp – was found. This finding suggests that treatments limiting Fe as well as Cu and Fe impose the accumulation of protein for the enzymes mediating Trp biosynthesis, regardless of transcript levels, although this response does not allow Trp accumulation in the double Cu and Fe deficiency. On the other hand, both ASP2 transcript and protein levels showed perfect synchronization (*r* = 0.99), increasing under both Fe and double Cu and Fe deficient conditions, although it did not explain the drop in Asp under the double deficiency (*r* = −0.55 and −0.65, respectively; Figure 4B and Table 2). Transcript levels of ASN2 exhibited the same pattern as Gln and Asn (*r* = 0.93 and 0.86, respectively), although protein levels tended to increase under single and double Cu and Fe deficiencies (Figure 4B and Table 2). Finally, ASPGA1 could only be investigated for transcript levels, which showed a good correlation with Asn concentration (*r* = 0.73; Figure 4B and Table 2). Collectively, our molecular analysis confirms that the specific drop in Trp and Asn concentration during simultaneous Cu and Fe deficient conditions cannot be explained by fluctuations in transcript or protein abundance of the enzymes participating in the biosynthesis of these amino acids.

**TABLE 2:** Correlation among transcriptome, proteome and metabolome changes in the tryptophan and asparagine biosynthesis pathways.

	RNA Prot	Trp (RNA/Prot)	Tyramine (RNA/Prot)		RNA Prot	Asp (RNA/Prot)	Gln (RNA/Prot)	Asn (RNA/Prot)
**PAI1**	NA	−0.64/NA	−**0.97**/NA	**ASP2**	**0.99**	−0.55/−0.65	**0.67**/0.57	**0.79**/**0.70**
**PAI2**	NA	−0.23/NA	0.39/NA	**ASN2**	0.22	0.48/−0.61	**0.93**/0.54	**0.86**/**0.67**
**InGPS**	−**0.87**	−0.58/0.18	−**0.97**/NA	**ASPGA1**	NA	−0.62/NA	0.60/NA	**0.73**/NA
**TSA1**	−**0.96**	−**0.84**/**0.76**	−**0.99**/**0.98**					
**TSB1**	**0.95**	0.24/0.49	0.24/0.49					

*Pearson correlation for pair-wise comparisons among transcript (RNA) and protein (Prot) abundance or metabolite content as indicated. Pearson correlation coefficients r > 0.65 in absolute value are highlighted in bold as relevant.*

**FIGURE 5:**
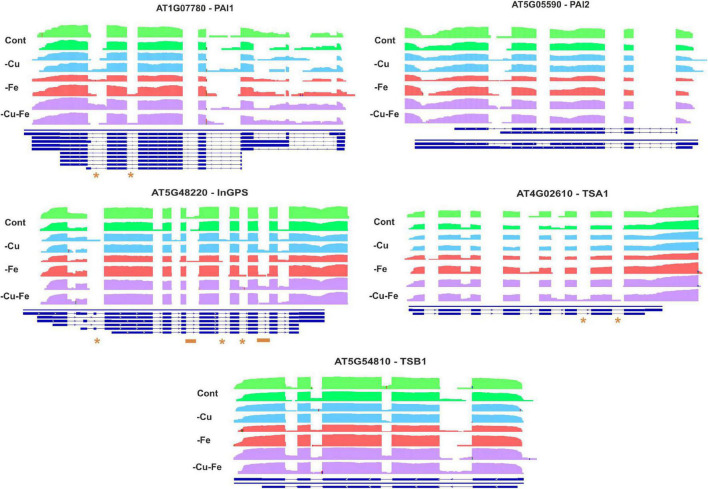
Visualization of alternative splicing events in the transcripts involved in tryptophan biosynthesis under single and simultaneous copper and iron deficiency. Log_10_-transformed read counts were used to represent the RNA-Seq coverage of intronic and exonic regions of PHOSPHORIBOSYLANTHRANILATE ISOMERASE 1 AND 2 (PAI1, 2), INDOLE-3-GLYCEROL PHOSPHATE SYNTHASE (InGPS), TRYPTOPHAN SYNTHASE BETA-SUBUNIT 1 (TBS1), TRYPTOPHAN SYNTHASE A (TSA1) under standard conditions (Cont) and single and double copper and iron deficiencies (–Cu, –Fe, and –Cu–Fe). Differences in the presence of intronic regions are depicted as asterisks (for changes in one replicate under –Fe or –Cu–Fe), or as orange lines (changes exclusive to –Cu–Fe conditions). Blue lines represent the gene model in TAIR10.

**FIGURE 6:**
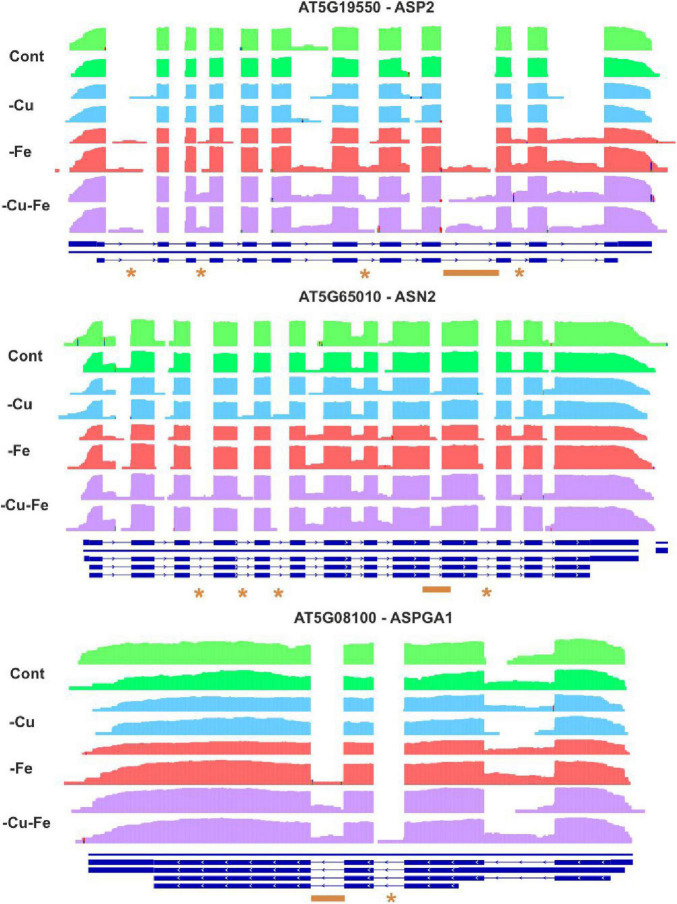
Visualization of alternative splicing events in the transcripts involved in asparagine biosynthesis under single and simultaneous copper and iron deficiency. Log_10_-transformed read counts were used to represent the RNA-Seq coverage of intronic and exonic regions of ASPARTATE AMINOTRANSFERASE 2 (ASP2), ASPARAGINE SYNTHETASE 2 (ASN2), and L-ASPARAGINASE (ASPGA1) under standard conditions (Cont) and single and double copper and iron deficiencies (–Cu, –Fe, and –Cu–Fe). Differences in the presence of intronic regions are depicted as asterisks (for changes in one replicate under –Fe or –Cu–Fe) or as orange lines (changes exclusive to –Cu–Fe conditions). Blue lines represent the gene model in TAIR10.

To further investigate the regulation of Trp and Asn biosynthesis, density plots were elaborated to visualize the RNA-Seq coverage of intronic and exonic regions of the transcripts of the above-investigated genes (Figures 5, 6). Intron bins harbored 1–12% of total coverage, depending on the gene and condition (Supplementary Figure 2). To track the most extreme differences, coverage maps were then manually curated to filter changes in AS events motivated by differential intron and exon composition from those due to differences in count abundance. As a general trend, Fe and double Cu and Fe deficiency led to differential coverage of intronic regions in at least one of the replicates. This is the case of *PAI1* (introns 5 and 6), *InGPS* (intron 8), *ASP2* (introns 1, 3, 7, and 10), and *ASN2* (intron 10) (see asterisks in Figures 5, 6). In three genes, the presence of additional introns was observed in only one replicate for the double Cu and Fe deficiency treatment, namely *TSA1* (introns 6 and 7), *ASN2* (intron 3), and *ASPGA1* (intron 2) (see asterisks in Figures 5, 6). Furthermore, we detected consistent IR events exclusively taking place under simultaneous Cu and Fe deficiency for *InGPS* (intron 6 and 9, Figure 5) in the Trp biosynthesis pathway and for all three selected enzymes for Asn biosynthesis, i.e., *ASP2* (intron 9), *ASN2* (intron 9), and *ASPGA1* (intron 3) (Figure 6). This observation suggests that the detrimental consequences related to Fe removal and, especially, the double Cu and Fe deficiency causes misfunctions in the AS machinery, which increases the frequency of different transcript isoforms due to IR events. Thus, the incapacity to increase Trp and Asn concentrations, albeit the high levels of biosynthetic enzymes under the double Cu and Fe deficiency, could, in part, be attributed to the accumulation of non-functional protein isoforms under this condition.

## Discussion

Transcriptome changes due to AS events in Arabidopsis adult rosette leaves exposed to single and double Cu and Fe deficiency for 10 days are not coincident with the fraction of transcripts changing in abundance. Even if 67.53% of the 2,637 DEGs reported in Garcia-Molina et al. (2020) could be potentially affected by AS, our analyses revealed that only 428 DEGs classified as DSGs for the interaction between Cu and Fe deficiencies (*p*-value ≤ 0.05, two-way ANOVA; Figure 1B). Previous studies also uncovered minimal overlap fractions between DEGs and DSGs in response to multiple nutrient deficiencies for 4 days, high salt, heat, or elevated CO_2_ levels (Ding et al., 2014; Nishida et al., 2017; Kannan et al., 2018; Huang et al., 2019), while Calixto et al. (2018) reported that almost half of the DEGs detected in plants cold-treated for 4 days underwent AS. Therefore, AS operates as an independent mechanism to refine plant responses to detrimental conditions beyond regulating gene expression.

In addition, changes in AS and transcript amounts resulting from the interaction between Cu and Fe deficiencies occurred according to different patterns. On the one hand, AS is a condition-dependent response mainly detected under Fe and double Cu and Fe deficiencies (Figure 1C). On the other hand, although specific changes in transcripts under Fe and simultaneous Cu and Fe deficiencies were found, the major fraction of transcript levels displayed an increase under Fe limitation that was more prominent during the double deficiency (Garcia-Molina et al., 2020). Per treatments, despite that Cu shortage for 10 days minimally impacted adult plants, we detected 200 transcripts exclusively undergoing AS under Cu deficiency, which is 10-fold the number of transcripts changing in abundance under this condition (Figure 1D). Fe deprivation provokes more profound physiological and molecular changes than Cu, but only imposed twice the number of Fe-specific DSGs than DEGs (262 *vs*. 125) (Figure 1D). However, Cu and Fe deficiencies applied simultaneously led to the major fraction of significant changes in AS events. Quantitatively, CuFe–DSGs were 2-fold the number of CuFe–DEGs (1,286 *vs.* 727) and 6- to 10-fold the number of DSGs found under single deficiencies, respectively (1,286 *vs.* 214 and 125) (Figure 1D). This finding provides more evidence supporting that combinatorial responses to simultaneous Cu and Fe deficiency trigger molecular mechanisms absent under single deficiencies.

In line with this, functional annotation of specific transcriptome changes related Fe-DEGs to Fe homeostasis and abiotic stress responses, whereas no significant terms for biological processes emerged when considering Cu–DEGs, Cu–DSGs, and Fe–DSGs. CuFe–DEGs enriched multiple terms, such as ribosome biogenesis, maturation of rRNA, response to cell cycle, oxidative stress, and both biotic and abiotic stimulus (Figure 2). However, CuFe–DSGs were involved in metabolic reactions, more precisely in amino acid biosynthesis. This fact indicates that general stress responses and adjustment in translational components during simultaneous Cu and Fe deficiency would be achieved by changes in transcript abundance, while certain aspects of plant metabolism might be systematically refined by AS.

Amino acids play a central role in the combinatorial response to simultaneous Cu and Fe deficiency in Arabidopsis. Compared with standard and Cu deficient conditions, amino acid pools tend to rise under Fe deficiency, already after 5 days of treatment, whereas under the double deficiency, this increase is more attenuated (Garcia-Molina et al., 2020). Metabolome profiling of Arabidopsis seedlings under double Cu and Fe deficiency uncovered a significant 2-fold increase in the amino acid fraction as a result of growth impairments and chloroplast misfunction. However, abrogation of Arabidopsis cytosolic fumarase FUMARASE2 (FUM2) conferred improved growth and photosynthesis rates under simultaneous Cu and Fe deficiency, and led to a barely unaltered amino acid composition in comparison with standard conditions (Garcia-Molina et al., 2021). Considering the interplay of Cu and Fe in sustaining antioxidant activities mediated by superoxide dismutases and electron transport chains in chloroplast and mitochondrion (Kliebenstein et al., 1998; Abdel-Ghany et al., 2005; Yamasaki et al., 2009; Bernal et al., 2012), the detrimental consequences of the simultaneous limitation of Cu and Fe would prevent the proper function of photosynthesis and respiration to obtain energy based on carbon skeletons. Given the potentiality of amino acids as energy sources (Hildebrandt et al., 2015), it could be likely that protein catabolism might act as a compensatory mechanism to fulfill plant energetic demands and, therefore, amino acid synthesis would be unnecessary under this scenario.

Having *Mapman* bins for central metabolism as a reference (Thimm et al., 2004), CuFe–DSGs retrieved genes coding for enzymes involved in Gln, Asp, Leu, Ser, Gly, Leu anabolism, and most of the central features in the Asn and Trp biosynthesis pathways (Table 1). Interestingly, Gln, Asn, and Trp appeared in the group of amino acids that rose in concentration under Fe deficient conditions and decreased or minimally increased during the double deficiency (cluster I, Figure 3). Moreover, Trp and Asn, as well as the Trp-derivative tyramine, Phe, fumaric acid, and adenine, were previously categorized as significantly altered metabolites in Garcia-Molina et al. (2020). To address regulatory mechanisms in the biosynthesis of metabolites changing in response to the combinatorial deficiency, the transcriptome, proteome and metabolome profiles previously reported were analyzed in combination. Single Fe and double Cu and Fe deficiency imposed similar changes in transcript and protein amounts for the selected enzymes involved in Trp and Asn biosynthesis, though not always coincident in trend (absolute *r* > 0.85; Figure 4 and Table 2). This indicates that the enzyme levels are not linearly dependent on the expression of the coding transcript. Indeed, enzyme amounts always increased under Fe and double Cu and Fe deficiency, regardless of the trend displayed by the coding transcript. However, the increase in enzyme levels could explain the increase in Trp and Asn concentration under Fe deficiency, but this is contradictory with the drop observed under simultaneous Cu and Fe deficiency. This observation could be attributed to either protein dysfunctionality, lack of intermediate availability, or faster consumption of the end products. Further analysis of the Trp pathway showed that tyramine – a degradative product of Trp – displayed a less dramatic decay under the double Cu and Fe condition in comparison with Trp. In that case, better correlations between tyramine concentration and enzyme amounts were found (*r* > 0.75), suggesting that tyramine would accumulate under the double deficiency as result of increased Trp degradation (Figure 4 and Table 2). On the other hand, Gln and Asp decreased in the same proportion as Asn under the combinatorial deficiency (Figure 4 and Table 2), ruling out the overaccumulation of metabolic intermediates caused by the drop in Asn.

Mechanistically, AS regulates gene expression either by the generation of transcript isoforms with altered stability, or by the production of modified protein versions with different functionality (Kelemen et al., 2013). In plants, IR is the most frequent AS event and has been well described as a phenomenon occurring during unfavorable conditions (Kalyna et al., 2011; Drechsel et al., 2013; Kwon et al., 2014; Filichkin et al., 2015; Ling et al., 2018). Although we found differences in AS motivated by differential count accumulation in introns, we described IR events in the Trp and Asn biosynthesis pathways under Fe and, especially, under double Cu and Fe deficiency (Figures 5, 6) that we attributed to misfunction of the splicing machinery due to the stress conditions imposed by the treatments. IR has been proposed to act as a strategy to promote transcript decay via the interruption of the reading frame (Kalyna et al., 2011; Drechsel et al., 2013), however, the biosynthetic enzymes for Trp and Asn that we analyzed increased at the protein level, regardless of the behavior of the coding transcript (Figure 4). Consequently, we proposed that the IR events taking place in the Trp and Asn biosynthesis pathways during the combinatorial response result in the codification of protein isoforms that are more stable, though less efficient in function. Nevertheless, we are not aware of any comprehensive study addressing the functional consequences of AS for the enzyme isoforms that we have described here. Similarly, the necessity of a specific regulation of Trp and Asn concentration during the double Cu and Fe deficiency remains elusive. Therefore, the role of such AS events as well as the changes in Asn and Trp concentration under the combinatorial Cu and Fe deficiency are open questions that deserve further investigation.

Taken together, our analysis sheds some new light on the systemic reprogramming of molecular changes that plants undergo during the combinatorial response to simultaneous Cu and Fe deficiency by including AS as an independent mechanism to regulate metabolic reactions that are not adjusted at either the transcript nor the protein level.

## Data Availability Statement

The original contributions presented in the study are included in the article/Supplementary Material, further inquiries can be directed to the corresponding author.

## Author Contributions

EM and AG-M designed the project, conducted the bioinformatic analysis, wrote the manuscript, and approved the submitted version.

## Conflict of Interest

The authors declare that the research was conducted in the absence of any commercial or financial relationships that could be construed as a potential conflict of interest.

## Publisher’s Note

All claims expressed in this article are solely those of the authors and do not necessarily represent those of their affiliated organizations, or those of the publisher, the editors and the reviewers. Any product that may be evaluated in this article, or claim that may be made by its manufacturer, is not guaranteed or endorsed by the publisher.

## Footnotes

1
https://cran.r-project.org/

2
https://bar.utoronto.ca/thalemine/

3
http://revigo.irb.hr/

4
https://mapman.gabipd.org/es_ES/home

## Supplementary Material

Supplementary Figure 1 Analysis of differential splicing events under individual and simultaneous deficiency of copper and iron. Venn diagrams for multiple comparisons among differentially splicing events per treatment according to an absolute fold-change of at least 2 in pair-wise comparisons (adjusted-*p*-value ≤ 0.05, two-way ANOVA with *post hoc* Tukey’s test).

Supplementary Figure 2 Primary analysis of intron retention in transcripts involved in tryptophan and asparagine biosynthesis under single and simultaneous copper and iron deficiency. The average of read counts per exon or intron bins for the selected genes under standard conditions (Cont) and single and double copper and iron deficiencies (–Cu, –Fe, and –Cu–Fe) was calculated using the *ASpli* package. Bars represent the percentage of reads detected in intronic bins over the total reads in the whole genic region (introns plus exons bins).
