# Supplementary figures and images for: Analysis of Alternative Splicing During the Combinatorial Response to Simultaneous Copper and Iron Deficiency in Arabidopsis Reveals Differential Events in Genes Involved in Amino Acid Metabolism
Source: Front Plant Sci. 2022 Jan 31;13:827828. doi: 10.3389/fpls.2022.827828 (PMC8841432; doi:10.3389/fpls.2022.827828)

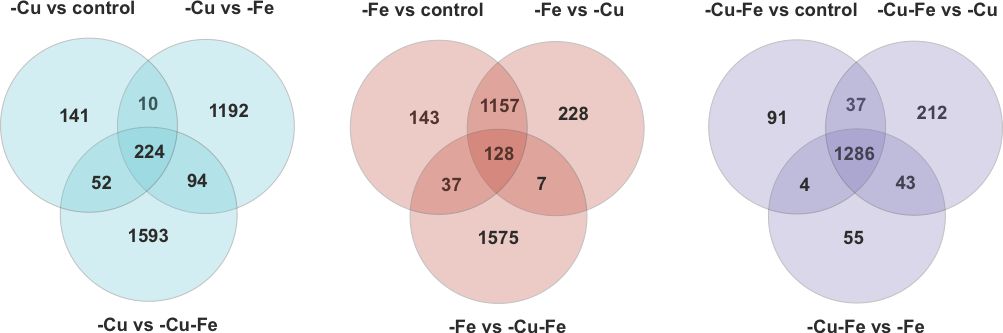

Supplement: Supplementary Figure 1 — Analysis of differential splicing events under individual and simultaneous deficiency of copper and iron. Venn diagrams for multiple comparisons among differentially splicing events per treatment according to an absolute fold-change of at least 2 in pair-wise comparisons (adjusted-p-value ≤ 0.05, two-way ANOVA with post hoc Tukey’s test). [file Image_1.JPEG]

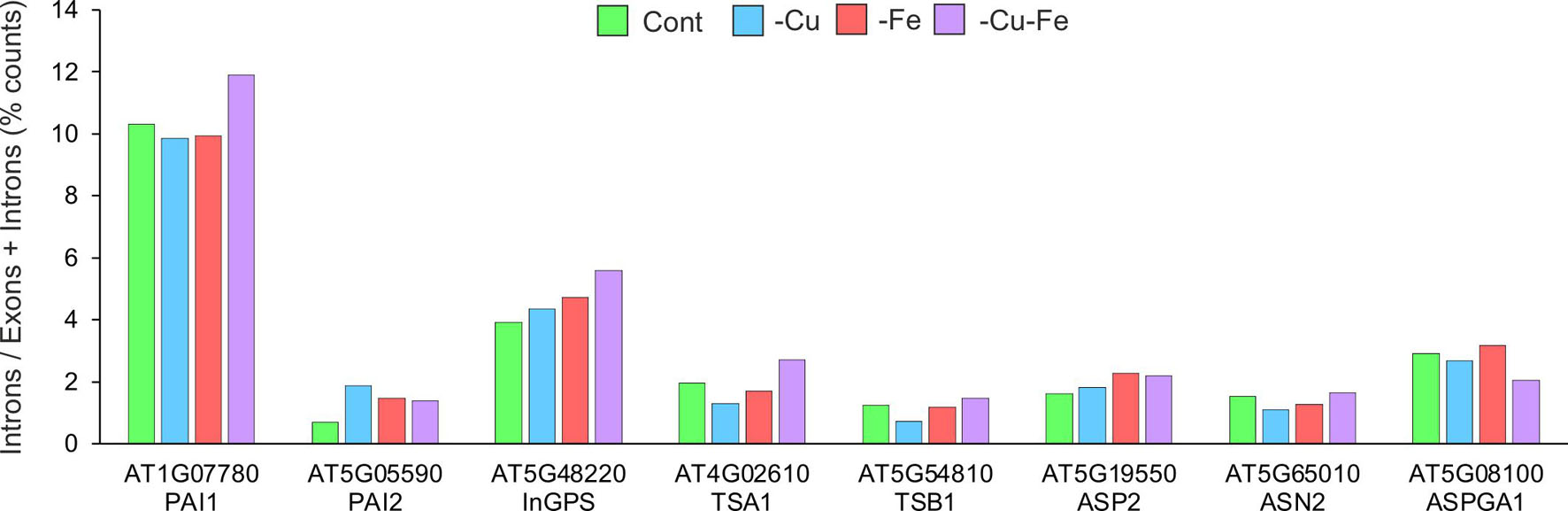

Supplement: Supplementary Figure 2 — Primary analysis of intron retention in transcripts involved in tryptophan and asparagine biosynthesis under single and simultaneous copper and iron deficiency. The average of read counts per exon or intron bins for the selected genes under standard conditions (Cont) and single and double copper and iron deficiencies (–Cu, –Fe, and –Cu–Fe) was calculated using the ASpli package. Bars represent the percentage of reads detected in intronic bins over the total reads in the whole genic region (introns plus exons bins). [file Image_2.JPEG]
